# Pregnancy‐specific dietary guidelines for Americans are not met: Findings from a pilot study

**DOI:** 10.1002/rfc2.63

**Published:** 2023-10-16

**Authors:** Noura El Habbal, Evgenia J. Filatava, Nicolette E. Overton, Matt Gregas, Katherine E. Gregory

**Affiliations:** ^1^ William F. Connell School of Nursing Boston College Chestnut Hill Massachusetts USA; ^2^ Department of Nursing Brigham and Women's Hospital Boston Massachusetts USA

**Keywords:** maternal health, pilot projects, pregnancy, pregnancy nutrition, prenatal care, term birth

## Abstract

**Objective:**

To assess maternal dietary intake during pregnancy and adherence to the 2020–2025 pregnancy‐specific Dietary Guidelines for Americans (DGA).

**Methods:**

This was a retrospective observational study. The study population consisted of women who gave birth to term infants (>37 weeks of gestation). Participants were given the Dietary Screener Questionnaire (DSQ) after birth and asked to recall their dietary intake in the last month of pregnancy. Participants' estimated dietary intakes were then compared to the 2020–2025 DGA which includes specific recommendations for pregnant women.

**Results:**

Out of 51 women who completed the DSQ, none consumed the recommended amounts of all surveyed dietary factors. Specifically, only one woman (2%) met the recommended intake of fruits, 11 women (22%) met the recommended intake of calcium, 25 women (49%) exceeded the recommended upper limit for added sugar intake, and none of the women (0%) met the intake of vegetables, whole grains, dairy and fiber.

**Conclusion:**

Women in our study did not adhere to the pregnancy‐specific DGA recommendations in the last month of pregnancy. Our findings underscore the need to increase maternal nutritional awareness and education to improve adherence to the DGA.

## Practitioner points

- None of the women in our study met the latest pregnancy‐specific 2020‐2025 Dietary Guidelines for Americans (DGA) recommendations for all dietary factors (vegetables, fruits, whole grains, dairy, calcium, fiber and added sugar) revealing suboptimal dietary intake in the last month of pregnancy.
- Prenatal nutritional counseling and education efforts are warranted to optimize maternal awareness and adherence to the DGA recommendations.
- Further assessment of current prenatal dietary intake is a public health priority to improve maternal and child health.

## INTRODUCTION

Maternal nutrition is considered a major factor influencing fetal development, birth outcomes and infant health.^1^, ^2^ Maternal undernutrition reduces fetal nutrient availability and is associated with low birth weight and an increased risk of infant cardiometabolic disease.^3^ Conversely, maternal overnutrition is associated with excessive gestational weight gain, fetal macrosomia, infant large for gestational age and childhood obesity which is a critical public health concern.^4^, ^5^ Although pregnant women tend to make healthier dietary choices throughout their pregnancy, most pregnant women do not meet the dietary recommendations due to a lack of awareness of dietary recommendations and suboptimal prenatal nutritional counseling.^6^, ^7^ Assessment of dietary intake of 125 pregnant women ages 20–44 years using 2013–2016 What We Eat in America, National Health and Nutrition Examination Survey (NHANES) 24‐h dietary recall data showed that most pregnant women consume diets that are low in fruits, vegetables, dairy and whole grains and are high in added sugars.^8^, ^9^ Furthermore, an analysis of overall diet quality among the same national samples of pregnant women revealed that they scored 63 out of 100 points on the Healthy Eating Index‐2015, suggesting that pregnant women fall short of meeting dietary guidance from the 2015–2020 Dietary Guidelines for Americans (DGA).^9^

The increased awareness of the importance of maternal diet on infant health outcomes has led the 2020–2025 DGA to include, for the first time, recommended dietary intake for pregnant women (by age group and trimester) and for lactating women.^10^ Although the addition of pregnancy‐specific dietary recommendations to the 2020–2025 DGA is an important public health milestone, these novel dietary recommendations warrant a more recent assessment of current maternal dietary intake to evaluate whether pregnant women adhere to the latest DGA. Our primary objective was to assess the dietary intake among pregnant women during the last month of pregnancy. Our secondary objective was to compare maternal dietary intake to the 2020–2025 DGA recommendations for women in the third trimester of pregnancy. Our exploratory pilot study may provide updated knowledge regarding maternal dietary intake and contribute to the promotion of prenatal nutritional counseling aimed at improving women's adherence to the DGA during pregnancy.

## MATERIALS AND METHODS

We conducted a retrospective observational pilot study evaluating maternal dietary intake during the last month of pregnancy among women who gave birth to term infants during one summer month (August 2021) at a large teaching hospital in Boston, Massachusetts, USA. This study was approved by the Mass General Brigham Human Research Committee (protocol #2016P001020) for which women provided written informed consent. No monetary compensation or other incentives were offered to participants.

The study inclusion criteria encompassed all women who gave birth to term infants (≥37 weeks of gestation). Women whose infants were admitted to the neonatal intensive care unit (NICU) during the postpartum hospital stay were excluded.

Maternal data were collected from available electronic medical records (Epic Systems Inc.). Maternal characteristics included race (American Indian/Alaska Native, Asian, Black or African American, more than one race, Native Hawaiian or other Pacific Islander, White, unknown or not reported), ethnicity (Hispanic or Latina, not Hispanic or Latina, unknown or not reported), age at the time of birth (years), prepregnancy body mass index (BMI;kg/m^2^), gestational age (weeks), gestational weight gain (kg), parity (nulliparous, multiparous), gestation type (singleton, twin pregnancy), mode of birth (vaginal, cesarean‐section), comorbidities and health conditions including diabetes (gestational diabetes mellitus or type 2), pre‐eclampsia/eclampsia, and chronic hypertension, and smoking status (nonsmoker, former smoker). Maternal race and ethnicity were self‐reported by the patients upon initial patient registration and are included here in a descriptive nature as they may be relevant in contextualizing social determinants of health.

We used a Dietary Screener Questionnaire (DSQ), which is a validated and rapid dietary screening tool. The DSQ was developed by the National Cancer Institute (NCI) as a time‐efficient method of dietary assessment that allows researchers to examine the intake of selected dietary factors within the past month^11^ and was validated by the 2009–2010 NHANES.^12^ The DSQ is convenient for use in a clinical setting,^13^, ^14^ such as during the brief postpartum hospitalization period immediately following birth, and it has been previously used to assess maternal dietary intake.^15^, ^16^ We decided to focus on dietary intake during the last month of pregnancy to reduce recall bias as the DSQ is uniquely developed to capture dietary intake over the past month. The DSQ contains questions relating to the consumption of food items that have a clearly demonstrated relationship with dietary factors (i.e., vegetables, fruits, whole grains, dairy, calcium, fiber, and added sugar)^17^ that align with the DGA. The DSQ was self‐administered and included instructions that asked participants to recall their diet and estimate the frequency of consumption of each food item within the past month. The DSQ responses were processed and converted into daily estimated intakes for seven dietary factors according to the publicly available DSQ scoring equations derived by the NCI.^18^ Then, the estimated daily intake for each dietary factor was compared to the dietary recommendations by the 2020–2025 DGA for pregnant women based on specific trimester and age group (i.e., third trimester and 31–50 years)^10^ to determine if women met the DGA recommendations or not as a binary outcome. Although DGA recommendations differ by maternal age (19–30 and 31–50 years) and trimester (first, second and third trimesters), the difference between age groups in the third trimester is minor. Furthermore, since the majority of women in our study were ≥31 years of age at the time of birth (*n* = 41, 80.4%) and all gave birth at term, we used the DGA recommendations for women ages 31–50 years in the third trimester of pregnancy as our reference. Of note, despite the DSQ containing two questions regarding the intake of processed and red meat, the NCI has not developed an algorithm to quantitatively assess the daily estimated intake of protein from the DSQ. Hence, we report maternal intake of processed and red meat as a binary qualitative outcome.

Descriptive analyses were used to summarize maternal characteristics. Continuous variables are reported as mean (standard deviation [SD] or confidence interval [CI]). Categorical variables are reported as absolute numbers; *n* (percentage; %). All analyses were performed using R statistical software (version 4.2.1).

## RESULTS

Out of 141 screened women, 52 (37%) women were approached and consented to participate in the study. The remaining 89 (63%) women were unavailable at the time of the approach, and were not approached at the request of the clinical team due to clinical circumstances at the time of the approach (e.g., spinal headache, extreme fatigue following labor and birth, lactation/breastfeeding), were found to be ineligible (i.e., NICU admission), or declined participation after being approached. Out of those who consented to participate in the study, 51 women completed the DSQ and one woman did not complete the DSQ before her hospital discharge. The average DSQ completion time was 1.4 ± 0.6 days postpartum (range 1–3 days). Maternal demographic and clinical characteristics are reported in Table 1.

**Table 1:** Maternal demographic and clinical characteristics (*n* = 51).

Characteristics	Mean (SD) or *n* (%)
Race	
Asian	5 (9.8%)
Black or African American	6 (11.8%)
Unknown or not reported	4 (7.8%)
White	36 (70.6%)
Ethnicity	
Hispanic or Latina	6 (11.8%)
Not Hispanic or Latina	45 (88.2%)
Maternal age at birth (years)	34.8 (3.7)
Maternal prepregnancy BMI (kg/m^2^)	26.7 (6.6)
Gestational age (weeks)	39.4 (1.0)
Gestational weight gain (kg)	13.5 (5.6)
Parity	
Nulliparous	18 (35.3%)
Multiparous	33 (64.7%)
Gestation type	
Singleton	48 (94.1%)
Twins	3 (5.9%)
Mode of birth	
Vaginal	29 (56.9%)
Cesarean section	22 (43.1%)
Maternal comorbidities	
None	45 (88.2%)
Pre‐eclampsia/eclampsia	1 (2.0%)
Chronic hypertension	2 (3.9%)
Diabetes (GDM or type 2)	3 (5.9%)
Former smoker	
No	44 (86.3%)
Yes	7 (13.7%)

Abbreviations: BMI, body mass index; GDM, gestational diabetes mellitus.

Maternal dietary intake in the last month of pregnancy and pregnancy‐specific DGA recommendations are detailed in Table 2. Of note, none of the participants met the 2020–2025 DGA recommendations for all seven dietary factors. Only 0%–2% of women met the recommended intake of fruits, vegetables, whole grains and fiber. Nearly a quarter of women (22%) met the recommended calcium intake, while almost half of the women (49%) exceeded the recommended upper limit for added sugar intake. The majority of women (92%) reported consuming processed or red meat during their last month of pregnancy.

**Table 2:** Maternal dietary intake during the last month of pregnancy (*n* = 51) compared to the 2020–2025 DGA recommendations for pregnant women.

Dietary factor	DGA recommended intake (third trimester; ages 31–50)	Participants' mean intake (CI)	Participants who met the DGA, *n* (%)
Vegetables (cup eq/day)	3	1.5 (1.4–1.6)	0
Fruits (cup eq/day)	2	1.1 (1.0–1.2)	1 (2%)
Whole grains (oz eq/day)	4	0.9 (0.8–1.0)	0
Dairy (cup eq/day)	3	1.7 (1.6–1.8)	0
Calcium (mg/day)	1000	905.9 (872.5–941.3)	11 (21.6%)
Fiber (g/day)	34	15.0 (13.8–16.2)	0
Added sugar (tsp eq/day)	<14.29	15.1 (13.6–16.6)	26 (51%)

Abbreviations: CI, confidence interval; DGA, Dietary Guidelines for Americans.

## DISCUSSION

Women in our study did not meet the 2020–2025 DGA recommendations for all seven dietary factors (vegetables, fruits, whole grains, dairy, calcium, fiber and added sugar) in the last month of pregnancy. Alarmingly, participants' intakes of individual dietary factors were also largely inadequate. These findings are in accordance with the most recent national data showing suboptimal dietary intake during pregnancy.^9^ According to the Scientific Report of the 2020 Dietary Guidelines Advisory Committee, which is based on the 2013–2016 NHANES data, 64% of pregnant women did not meet the 2015–2020 DGA recommended fruit intake, 90% did not meet vegetable intake, 95% did not meet whole grain intake, 90% did not meet dairy intake and 99% exceeded the maximum limit of energy from solid fats and added sugars.^9^ Similar trends have also been reported in more recent publications. For example, in a secondary analysis which included 847 women drawn from a large national cohort, dietary intake assessed by a modified diet history questionnaire, revealed that 44% of pregnant women did not meet the 2015–2020 DGA recommended vegetable intake.^19^ In a randomized clinical trial by Hull et al., fiber intake was assessed by 24‐h recalls among a sample of eight pregnant women in the control group and revealed an average intake of around 17 g/day,^20^ which does not meet the 2020–2025 DGA recommendations. Interestingly, Hull et al. showed that fiber intake increased to an average of around 27 g/day in the treatment group, which included eight pregnant women who received educational lessons focused on the benefits of consuming a high‐fiber diet.^20^ This suggests that prenatal nutritional counseling and knowledge of the DGA may be important factors in promoting a healthy dietary pattern during pregnancy.

Strengths of our study include having our data collected during the summer, which eliminates potential seasonal dietary changes and reflects a period when fruits and vegetables are more readily available and accessible in the Northeastern United States. Furthermore, when compared to the common dietary assessment tools (i.e., 24‐h dietary recall and food frequency questionnaires), the DSQ is a convenient, time‐efficient and validated questionnaire that reduces patient burden and minimizes disruptions in the clinical care required during the immediate postpartum period. Finally, our exploratory study is relevant and timely as our data were collected soon after the publication of the first‐ever pregnancy‐specific DGA recommendations, and as such, our findings may contribute to an initial foundation from which future studies on diet and health during pregnancy can build.

Some limitations of our study include having a small sample size, providing a snapshot of maternal dietary intake during the last month of pregnancy, and limiting our recruitment to a single hospital thus affecting the generalizability of our findings to a broader population. Additionally, the mean maternal age in our study was 34.8 years, which may not be generalizable to all pregnant women. Future research with larger and more diverse study populations is needed to increase the statistical power and generalizability of the results. Furthermore, given our sample size, we were unable to assess dietary intake as a function of health conditions (e.g., diabetes, chronic hypertension and pre‐eclampsia/eclampsia), gestation type, prepregnancy weight, or gestational weight gain in a meaningful manner. Our descriptive study also does not contain important sociodemographic or socioeconomic factors such as employment status, insurance status, household income, paid maternity leave availability, education, food access or insecurity and spoken languages, which can all influence dietary intake, purchasing power and food choices.^19^, ^21^, ^22^ Additionally, our study only includes term infants, which does not allow us to examine the associations between maternal diet and pregnancy outcomes (e.g., preterm birth). Future work can evaluate the impact of maternal nutrition, in light of the newest DGA recommendations, on infant gestational age at birth, birth weight and growth outcomes. In regard to the DSQ, it is also important to emphasize that despite the validation of this questionnaire, it is self‐reported and subject to participant recall bias, which is a common limitation of several dietary assessment methods. The DSQ also does not have a formal quantitative measure of protein and fat intake and does not allow for reporting of exact portion sizes, thus limiting our ability to assess the dietary intake of all dietary factors and their exact amounts. Future studies can assess dietary intake using more elaborate tools such as the food frequency questionnaire or 24‐h recalls throughout the entire pregnancy (first, second and third trimesters) to comprehensively capture maternal dietary intake and compare it to the trimester‐specific DGA recommendations.

## CONCLUSION

Our findings highlight the inadequacy of dietary intake during the last month of pregnancy among women who gave birth to term infants. In our study population, we found that most women did not meet the 2020–2025 DGA recommendations of major dietary factors. Our study reinforces the need to raise awareness about following a healthy diet during pregnancy, the new pregnancy‐specific DGA, and the importance of nutritional education and intervention on the part of the multidisciplinary team caring for pregnant women and seeking to optimize maternal health. Our findings indicate that more research is needed to better understand maternal dietary intake and adherence to the first‐ever pregnancy‐specific DGA recommendations and to provide additional data that could help inform the next iteration of the pregnancy‐specific DGA. Future research on this topic could also provide evidence that informs prenatal nutritional education and counseling and may lead to policy‐level interventions that improve maternal dietary intake.

## AUTHOR CONTRIBUTIONS

Evgenia J. Filatava, Nicolette E. Overton and Katherine E. Gregory designed and conceptualized research. Evgenia J. Filatava and Nicolette E. Overton conducted research. Noura El Habbal and Matt Gregas analyzed data. Noura El Habbal and Evgenia J. Filatava wrote the paper. Katherine E. Gregory had primary responsibility for the final content. All authors read and approved the final manuscript.

## CONFLICT OF INTEREST STATEMENT

The authors declare no conflict of interest.

## ETHICS STATEMENT

This study was approved by the Mass General Brigham Human Research Committee (protocol # 2016P001020).

## Data Availability

The data supporting the findings of this study are available from the corresponding author upon reasonable request.
